# P-813. Lactobacillus Bacteremia and Endovascular Infections: A Retrospective Case Series of 333 Patients

**DOI:** 10.1093/ofid/ofae631.1005

**Published:** 2025-01-29

**Authors:** Christopher Radcliffe, Melis N Anahtar, Elizabeth Hohmann, Sarah Turbett, Caitlin Dugdale, Molly Paras

**Affiliations:** Massachusetts General Hospital, Boston, Massachusetts; Mass General Hospital, Cambridge, Massachusetts; Massachusetts General Hospital, Boston, Massachusetts; Massachusetts General Hospital, Boston, Massachusetts; Massachusetts General Hospital, Boston, Massachusetts; Massachusetts General Hospital

## Abstract

**Background:**

Despite being normal flora, and sometimes considered a contaminant, there are reports of *Lactobacillus* endovascular infections. However, large case series are lacking. We evaluated patient characteristics, antimicrobial susceptibility testing (AST), and all-cause mortality among patients with *Lactobacillus* bacteremia and/or endovascular infections managed in the Mass General Brigham (MGB) healthcare system.

**Table 1.**
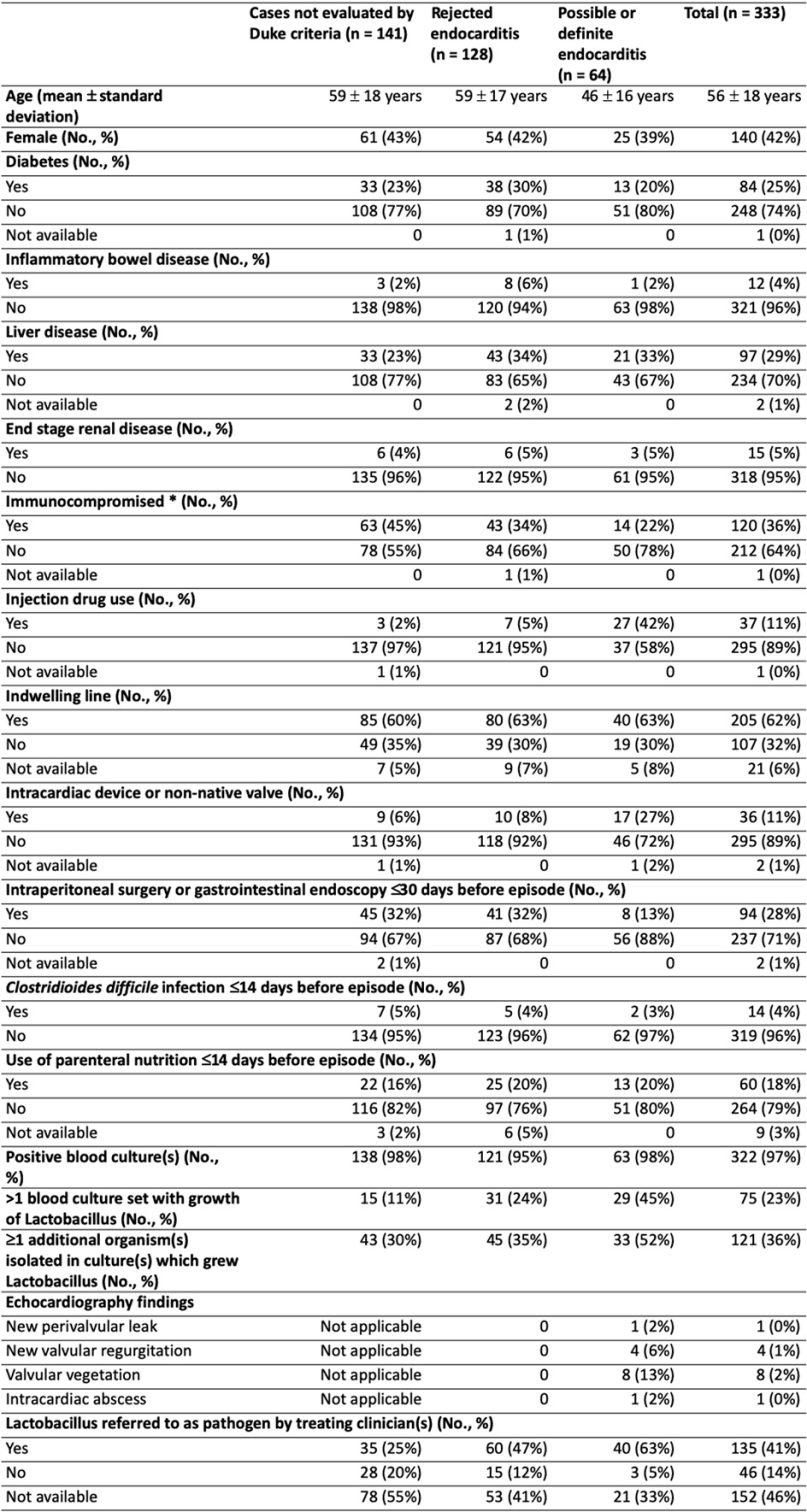
Patient Characteristics for Lactobacillus Bacteremia and Endovascular Infections * Immunocompromised based on one or more of the following diagnoses: a. Human immunodeficiency virus with CD4 <200 b. Active lymphoma or leukemia (including indolent chronic lymphocytic leukemia) c. Metastatic cancer d. Cytotoxic chemotherapy within the prior 3 months e. Radiation therapy within prior 3 months f. Congenital immunodeficiency g. Aplastic anemia h. Solid organ transplant recipients on immunosuppressive therapy i. Hematopoietic stem cell transplant recipients, unless >2 years post-transplant and no longer on any immunosuppressive therapy j. Immunocompromised based on >1 of the following medications: glucocorticoid therapy equivalent to prednisone >20 mg/d for >2 weeks, or if such therapy has been discontinued within the past month; alkylating agents within the past 3 months; antimetabolites (methotrexate >0.4 mg/kg/week, azathioprine >3 mg/kg/day, 6-mercaptopurine >1.5 mg/kg/day) within the past 3 months; cyclosporine, tacrolimus, sirolimus, everolimus, or mycophenolate mofetil within the past 3 months; biologic immunosuppressants and immunomodulators within the past 3 months (6 months for lymphocyte-depleting agents)

**Methods:**

The MGB Research Patient Data Registry and Massachusetts General Hospital microbiology database were queried to identify adults with blood, central line, or endovascular device culture(s) positive for *Lactobacillus* obtained at in-network hospitals between 1/1/00 and 9/1/22. Patient demographics, comorbidities, antibiotic regimens, 90- and 365-day all-cause mortality, and AST data for *Lactobacillus* isolates were extracted from charts when available. When echocardiography was available, potential endocarditis was classified according to the 2000 Duke criteria. Summary statistics were performed.

**Table 2.**
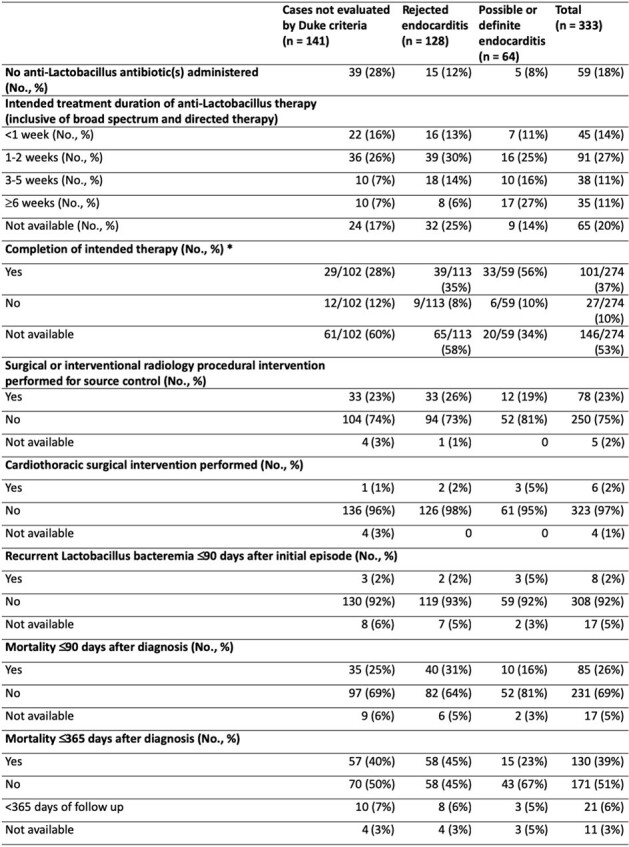
Treatment Strategies and Outcomes for Lactobacillus Bacteremia and Endovascular Infections *Number of cases reported to have completed intended therapy does not include cases for which no anti-Lactobacillus therapy was administered

**Results:**

We identified 333 patients with *Lactobacillus* bacteremia and/or endovascular infections. The mean age was 56 years, 42% (140/333) were female, and 36% (120/333) were immunocompromised (**Table 1**). Possible or definite endocarditis was identified in 19% (64/333), and 36% (121/333) had ≥1 additional organism(s) isolated in culture(s) growing L*actobacillus*. The most common intended treatment duration was 1-2 weeks (91/209; 44%). Overall, 59/333 (18%) patients did not receive antibiotics active against *Lactobacillus* (**Table 2**). For patients with data available, all-cause mortality was 27% (85/316) at 90 days and 43% (130/301) at 365 days. For isolates with AST data, susceptibility to penicillin, ampicillin, and linezolid was reported for 96% (82/85), 99% (76/77), and 100% (66/66), respectively. Most isolates were resistant to meropenem (40/49; 82%) (**Table 3**).

**Table 3.**
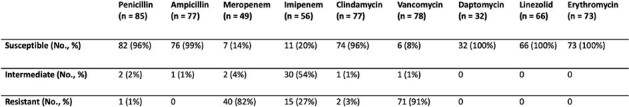
Antimicrobial Susceptibility Data for Unique Lactobacillus Isolates

n reflects number of unique Lactobacillus isolates that were tested against each antimicrobial

**Conclusion:**

Among 333 patients with *Lactobacillus* bacteremia and/or endovascular infections, 1-year all-cause mortality was high. When *Lactobacillus* is isolated in blood cultures, clinicians should consider its relevance to the clinical scenario before labeling the organism as a contaminant.

**Disclosures:**

**Melis N. Anahtar, MD, PhD**, Day Zero Diagnostics: Advisor/Consultant|Day Zero Diagnostics: Ownership Interest **Elizabeth Hohmann, MD**, Astra Zeneca: Grant/Research Support|Laguna Biotherapeutics: Grant/Research Support|MicrobiomeX/Tend: Grant/Research Support **Sarah Turbett, MD**, UpToDate: Author

